# Characterization and comparison of strains of *Pasteurella multocida* associated with cases of progressive atrophic rhinitis and porcine pneumonia in Argentina

**DOI:** 10.14202/vetworld.2019.434-439

**Published:** 2019-03-21

**Authors:** Fernando A. Bessone, Maria Laura Soriano Pérez, Gustavo Zielinski, Marina Dibarbora, M. B. Conde, Javier Cappuccio, Fabrisio Alustiza

**Affiliations:** 1 Department of Animal Health, Instituto Nacional de Tecnología Agropecuaria, Estación Experimental Agropecuaria Marcos Juárez, Marcos Juárez, Córdoba, Argentina; 2 CONICET, Buenos Aires, Argentina

**Keywords:** antibiotics susceptibility profiles, molecular characterization, *Pasteurella multocida*, pig

## Abstract

**Background:**

*Pasteurella multocida* (Pm) is the causative agent of progressive atrophic rhinitis (PAR) and pneumonic pasteurellosis (PN) in pigs. Pm is a member of the porcine respiratory complex responsible for important economic loss in the pig industry.

**Aim:**

This study aimed to characterize the Pm strains recovered from clinical cases of PN and PAR and to elucidate the antibiotic susceptibility profiles of the strains.

**Materials and Methods:**

Sixty strains were characterized molecularly by polymerase chain reaction to determine species-specific gene, capsular type (A or D), and toxin A production. The agar diffusion method was employed to evaluate antibiotic resistance profiles.

**Results:**

We found that 65% of strains belonged to capsular type A or D, and 15% of those were positive to *toxA* gene. The antibiotic susceptibility profiles found were sensitive in decreasing order to: Enrofloxacin, ceftiofur (CTF), ampicillin, tilmicosin (TIL), florfenicol (FFN), spectinomycin (SPC), gentamicin, oxytetracycline (OTC), and trimethoprim-sulfamethoxazole (TMS). Strains were resistant in decreasing order to: Lincomycin (LIN), tylosin (TYL), erythromycin (ERY), TMS, SPC, OTC, FFN, TIL, and CTF.

**Conclusion:**

The *toxA* gene was detected in many Pm isolates from pneumonic lungs. Capsule type A or D was the most frequently found among the collected isolates. LIN, TYL, and ERY are the drugs which showed higher percentages of resistant isolates.

## Introduction

Progressive atrophic rhinitis (PAR) and pneumonic pasteurellosis (PN) in pigs are the main diseases caused by *Pasteurella multocida* (Pm) [1]. This agent is part of the porcine respiratory complex (PRC) and is responsible for significant economic losses at the productive level in porcine farms [2].

Numerous critical genes for Pm virulence have been identified in an attempt to elucidate the pathogenesis of pasteurellosis, which remains as a puzzle in many of its host species such as pigs [3-8]. The capsule is a key virulence factor in the pathogenesis of pasteurellosis in pigs. It has been established, by construction of genetically defined mutants, unable to synthesize or export the capsule to its surface, that virulence of original strains was attenuated [9,10]. The identification of capsid biosynthesis genes (*capA*, *capB*, *capD*, *capE*, and *capF*) led to the development of DNA-based typing by polymerase chain reaction (PCR) technique [9,10], an alternative to the serological methods traditionally employed (serogroups A, B, D, E, and F based on the antigenicity of their polysaccharide capsule) [11-13]. The dermonecrotic toxin of Pm is a 146 kDa protein encoded by the *toxA* gene responsible for the clinical signs and pathogenesis of PAR. The toxigenic strains associated with PAR frequently belong to capsular type D although toxigenic isolates of capsular type A may also be involved in this disease [14-16]. In contrast to isolates associated with PAR, Pm isolated from PN is generally non-toxigenic capsular type A strains [1,4]. However, small proportions of strains isolated from lungs resulted to be toxigenic and/or belonged to capsular type D [16,17]. Description of pneumonia outbreaks caused by Pm in India, affecting various categories of pigs in a group of farms, is available [18]. Information about the circulation and characterization of Pm among pigs in Argentina [13,19] is limited and outdated. The emergence of antibiotic multiresistant Pm strains implies the need to carry out permanent surveillance of antimicrobial susceptibility profiles [20]. To determine the antibiotic resistance profiles of autochthonous swine, Pm isolates in Argentine is a permanent challenge. Therefore, updated information about circulating strains and its susceptibility to antibiotics is necessary to plan adequate control measures against the diseases produced by the species.

This study aimed to characterize the attributes of virulence and the antimicrobial resistance profiles expressed by a collection of Pm strains recovered from clinical cases of PN and PAR and to elucidate the antibiotic susceptibility profiles of the strains.

## Materials and Methods

### Ethical approval

To guarantee the correct and careful handling of pigs, investigators proceeded according to specifications of Ethical Guidelines (Internal Ethical Committee CICUAE Res. 533/16).

### Sampling

Nasal swabs and lung samples (n=115) both from diseased animals were received in the Animal Health Laboratory of Instituto Nacional de Tecnología Agropecuaria (INTA) Estación Experimental Agropecuaria Marcos Juarez, in the period 2010-2014. All samples were taken from commercial intensive farms of the Province of Cordoba in the central region of Argentina.

### Bacterial isolates

We analyzed 60 isolates of Pm recovered from the samples, which were collected through culturing nasal swabs and lung samples from pigs with clinical cases of PAR and pneumonia, respectively. Samples were inoculated onto 6% equine blood agar plates, incubated at 37°C for 24 h, and identified by Gram staining, morphology (bacilli or coccobacilli), and biochemical test: Oxidase (positive), SIM (indole positive and non-motile), and urease (negative).

### DNA purification

DNA from identified strains was extracted using commercial kits (Qiagen^®^), according to manufacturer’s instructions. Samples were stored at −20°C. These extracts were used as templates for PCR reaction.

### Molecular characterization

All Pm isolates (n=60) were analyzed by PCR to characterize the following traits: Species-specific gene of Pm and capability to produce toxin type A and capsular type (A and D). In Table-1, the gene primers used are listed [21,22]. The PCR mixture was composed of 5 µl of DNA 50 µM of each primer (Fagos^®^), 100 µM dNTPs (Promega), 1.5 µM of MgCl, 1X buffer (5X-Go Taq Promega^®^). and 0.5 U of Taq Polymerase (Promega^®^) in a final volume of 25 µL. Reactions were subjected to one initial denaturalization cycle of 94°C for 60 s, followed by 30 cycles of 30 s at 94°C denaturation, 30 s at 58°C (primers annealing), 30 s at 72°C (extension), and finally, 2 min at 72°C (final extension). Reference strains of Pm were used as pathotype and positive control reaction (Table-1). For revealing the amplified products, standard electrophoresis in 1% of agarose in Tris-acetate EDTA buffer stained with ethidium bromide performed at 100V during 30 min was used. Visualization of amplicons was made under transilluminator (UV-λ 300nm), and the 100 bp ladder was used. Gels were photographed with a Kodak Easy Share Z7590 camera system and evaluated with a Kodak Digital Science 1D software.

**Table-1:** Primers sequences of Pm virulence factor.

Virulence factor	Description	Primer ID	Sequence 5×-3×	Amplicon size (bp)	References
*ToxA*	Toxin A	*TOX-FWD*	CGTGAACTGCGTACTCAA	1854	[22]
		*TOX-REV*	AAGAGGAGGCATGAAGAG		
*KMT1*	Species-specific gene	*KMT1T7*	ATCCGCTATTTACCCAGTGG	460	[21]
		*KMT1SP6*	GCTGTAAACGAACTCGCCAC		
*A hyaD-hyaC*	Capsular type A	*CAPA-FWD*	TGCCAAAATCGCAGTCAG	1044	[22]
		*CAPA-REV*	TTGCCATCATTGTCAGTG		
*D dcbF*	Capsular type D	*CAPD-FWD*	TTACAAAAGAAAGACTAGGAGCCC	657	[22]
		*CAPD-REV*	CATCTACCCACTCAACCATATCAG		

### Antibiotic profiles

A disk diffusion method was performed according to recommendations of the National Committee for Clinical Laboratory Standards (NCCLS, 2014) [23]. Briefly, 12 different antibiotics were assayed: Enrofloxacin (ENR), ceftiofur (CTF), ampicillin (AMP), tilmicosin (TIL), erythromycin (ERY), lincomycin (LIN), spectinomycin (SPC), florfenicol (FFN), gentamicin (GEN), trimethoprim-sulfamethoxazole (TMS), oxytetracycline (OTC), and tylosin (TYL). The manufacture guidelines were used for inhibition halo interpretation. Multiple antibiotic resistance was defined as isolates, showing three or more antimicrobial classes resistance.

### Statistical analysis

To evaluate associations between variables (antibiotic profiles), conglomerates analysis was employed with Euclidea’s variables definition, to know the levels of variable relation. InfoStat version 2015 [24] software was employed (InfoStat^®^, National University of Córdoba, Argentina).

## Results

**Figure-1:**
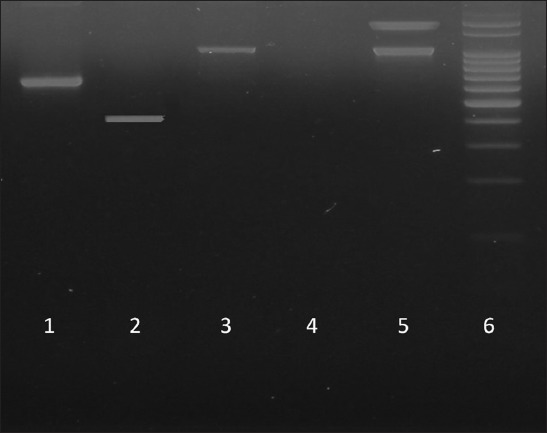
Electrophoresis gel of polymerase chain reaction products. Lane 1: *CapD*; Lane 2: *KMT1*; Lane 3: *CapA*; Lane 4: *Negative control*; Lane 5: *CapD*; Lane 6: *Ladder* (*100bp*).

The 60 strains studied were positive for the species-specific PCR of Pm (Figure-1). It was observed that 65% of the isolates were capsular type A (32.5%) or D (32.5%), while the remaining 35% belonged to other capsular types (not determined in this work). It was also observed that 16.6% of the studied strains were amplified for the *ToxA* gene (Table-2), beyond the associations with pathologies studied.

**Table-2:** Association of capsular types, presence of toxin, and pathogenicity of Pm isolates.

Strain ID	Type A	Type D	Cap. ND	Tox A	PN	PAR
Pm001	+	−	−	−	+	−
Pm002	−	+	−	−	−	+
Pm003	−	−	+	−	−	+
Pm005	+	−	−	+	+	−
Pm006	−	+	−	−	−	+
Pm008	+	−	−	−	+	−
Pm010	+	−	−	−	+	−
Pm011	−	+	−	−	−	+
Pm012	-	+	-	-	-	+
Pm014	−	−	+	−	+	−
Pm017	+	−	−	−	+	−
Pm019	−	−	+	+	+	−
Pm020	−	+	−	+	−	+
Pm021	+	−	−	−	+	−
Pm025	+	−	−	−	+	−
Pm026	−	−	+	−	−	+
Pm028	+	−	−	+	+	−
Pm029	+	−	−	−	+	−
Pm031	−	+	−	−	−	+
Pm032	+	−	−	−	+	−
Pm038	−	−	+	−	−	+
Pm040	−	+	−	−	−	+
Pm041	−	+	−	+	−	+
Pm042	−	+	−	−	−	+
Pm043	−	+	−	−	−	+
Pm044	−	+	−	−	−	+
Pm048	−	+	−	−	−	+
Pm049	−	+	−	−	−	+
Pm052	+	−	−	+	+	−
Pm053	−	−	+	−	+	−
Pm054	−	+	−	−	−	+
Pm061	+	−	−	−	+	−
Pm063	+	−	−	−	+	−
Pm066	-	+	-	-	-	+
Pm073	−	−	+	−	+	−
Pm074	−	−	+	−	+	−
Pm075	+	−	−	−	+	−
Pm076	−	−	+	−	+	−
Pm079	−	+	−	−	−	+
Pm080	−	+	−	−	−	+
Pm084	−	−	+	−	+	−
Pm086	−	−	+	−	−	+
Pm093	−	−	+	+	+	−
Pm095	−	−	+	−	+	−
Pm097	−	−	+	+	+	−
Pm098	−	+	−	−	−	+
Pm099	−	−	+	−	−	+
Pm100	−	−	+	−	+	−
Pm101	+	−	−	−	+	−
Pm103	+	−	−	−	+	−
Pm104	+	−	−	−	+	−
Pm106	−	−	+	−	+	−
Pm107	+	−	−	−	+	−
Pm108	−	−	+	−	+	−
Pm109	−	−	+	−	+	−
Pm110	+	−	−	−	+	−
Pm111	−	−	+	+	+	−
Pm112	−	+	−	−	−	+
Pm113	−	−	+	+	+	−
Pm114	−	−	+	−	+	−
Total (%)	19 (32.5)	19 (32.5)	22 (35)	10 (16.6)	36 (60)	24 (40)

References - Pm=*Pasteurella multocid*a, PN=Pneumonic pasteurellosis, PAR=Progressive atrophic rhinitis, Cap ND=Capsular type not determined

**Figure-2:**
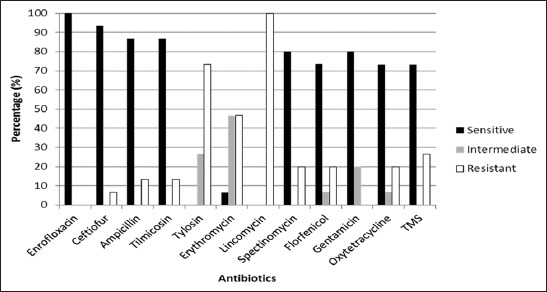
Profiles of antibiotic susceptibility of *Pasteurella multocida* strains.

In relation to the antibiotic susceptibility profiles, about 70% of strains showed sensitivity to ENR, CEF, AMP, TIL, FFN, SPC, GEN, OTC, and TMS (Figure-2). All strains were resistant to LIN, 70% of strains showed resistance to TYL, while resistance to CEF (7%), AMP (13%), TIL (13%), ERY (48%), SPC (20%), FFN (20%), OTC (20%), and TMS (27%) was also found. Antibiotics tested are frequently used in commercial intensive farms.

Multiple resistance profiles to antibiotics were detected. Association between resistance profile and pathological condition is shown in Table-3.

**Table-3:** Prevalent multiresistance profile.

Multi-resistance profile	Frequency* n (%)	Pathological condition	

		PAR (n)	PN ((n)
Tilmicosin–Tylosin–Erythromycin	10 (17)	7	3
Tilmicosin–Tylosin–Lincomycin	10 (17)	6	4
Tilmicosin–Erythromycin–Lincomycin	10 (17)	6	4
Tylosin–Erythromycin–Lincomycin	33 (56)	14	19
Tylosin–Lincomycin–TMS	5 (8.5)	2	3

References -

* These values are not additives. PAR=Progressive atrophic rhinitis, PN=Pneumonic pasteurellosis, TMS=Trimethoprim sulfamethoxazole

**Figure-3:**
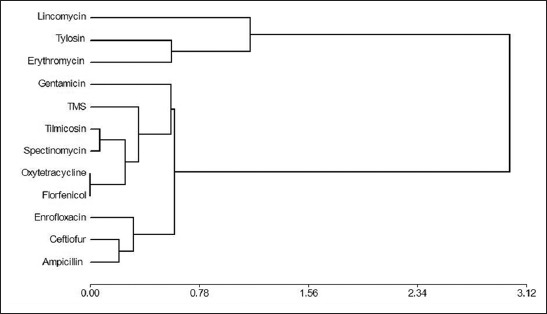
Dendrogram shows conglomerates analysis of antibiotic profiles of *Pasteurella multocida* from progressive atrophic rhinitis and pneumonic pasteurellosis cases.

Strains isolated from animals with both pathological conditions, PAR and PN, showed extended antibiotic multi-resistance profiles (conglomerate analysis) (Figure-3).

To analyze the profiles of resistance of isolates, a dendrogram was constructed. As shown in Figure-2, two principal groups forming two conglomerates were found, with a distance of practically two points. The conglomerate constituted by LIN (100%), TYL (70%), and ERY (48%) are drugs with the highest percentage of resistance. The other associations of conglomerate cluster of resistance, intermediate susceptibility, and sensitivity, in descending order from top to bottom, are graphed.

## Discussion

Isolation of Pm from PRC is a relatively frequent event. However, updated information about characteristics of strains recovered in our country in terms of toxicity, capsular type, or antibiotic resistance profiles is limited. The present work provides relevant information about these topics from a collection of strains isolated from pigs affected by PN or PAR in farms from Argentina.

The PCR technique turned out to be rapid and reliable to determine the capsular types A and D as well as Pm species-specific gene (*KMT1*), similarly to previously reported by other authors [9,10,16,21].

Our results showed that, from 36 isolates associated with PN, only 19 (53%) resulted as being of capsular type A. Even more, from 24 isolated strains from animals affected by PAR, 19 (83%) were found to be capsular type D. This association, in terms of disease symptoms, macroscopic lesions, and capsular type, for both diseases is consistent with previous studies where cases of PN were related to capsular type A and cases of PAR were related to capsular type D [1,16].

The *toxA* gene was detected in eight isolates associated with PN and only in two isolates associated with PAR; these findings do not agree with previous studies [15,25], reporting that toxigenic type A Pm is the main causative agent of nasal turbinate atrophy in swine. In the present investigation, seven strains positive to *toxA* were isolated from lungs associated with PN; however, turbinates from pneumonic animals were not inspected so that there is a chance that those animals could have been also affected by PAR. Although the role of Pm as the primary agent of respiratory infections in PRC is still discussed and unclear, the present findings provide some evidence concerning involvement of toxigenic Pm in PN. The old debate is still open: Could Pm play a primary etiological role in the pathogenesis of porcine pneumonia, not always behaving as an opportunistic pathogen as cited in other studies? [25]. We considered necessary to answer that question, a greater number Pm strains from PN be studied in order to deepen the knowledge about the role of this pathogen. Isolation from PAR cases as well as from PN should be collected and studied, and also, experimental infection models should be mounted to correlate the isolate genetic expression to host disease production.

We considered highlighting the importance of maintaining continuous surveillance of antibiotic susceptibility profiles of pathogens such as Pm. This is so because of the limited availability of the menu of therapeutic agents that are active and whose usage is legal to neutralize microorganisms causing PRC. In this work, ENR was the unique antibiotic with antimicrobial activity against 100% of the strains tested, followed by CTF (93.3%), AMP (86.7%), TIL (86.7%), and GEN (80%). Similar findings were reported by Tigga *et al*. [18] in India, who found 100% of sensitivity to ENR and GEN; however, TMS and OTC results were different. Susceptibilities of 100% of strains tested to AMP, CTF, ENR, FFN, tilmicosin, and penicillin were communicated in the USA and Canada [20]. In contrast, we found a higher proportion of resistant strains to ampicillin (13.3%), FFN (13.3%), and TIL (20%) but more susceptible strains to OTC (73.3%). In regard to previous work in our country, about 13 Pm isolates were found to be susceptible to ampicillin, CTF, ENR, FFN, GEN, tiamulin, and tetracycline [13]. Moredo *et al*. [19] found percentages of around 35% of strains susceptible to ampicillin, tetracycline, ENR, and FFN. Our findings partially agree with these reports providing updated information concerning the matter. It is possible that the resistance to TYL found in our work could be due to the massive usage of this drug in the feed. TYL is commercially available, being easy to handle through the food ration. Even more, it was extensively used in respiratory and digestive problems, as well as a growth promoter. Although antibiotics with a broad spectrum of activity have been registered, we considered important to remark the increase of resistance and intermediate resistance developed by some strains.

## Conclusion

In the central region of Argentina, 15% of the studied strains, many of which were isolated from lungs with diagnosis compatible with PN, possess the *ToxA* gene. Antibiotic resistance profiles showed that LIN, TYL, and ERY are drugs with a higher percentage of resistance among strains tested.

Knowledge of the phenotypic characteristics of strains of Pm isolated in Argentina is important for better control of the diseases produced by the organism; however, it would be necessary to analyze a greater number of strains within the framework of epidemiological studies.

## Authors’ Contributions

Conceived and designed the experiments: FAB, FA, MD, JC, and GZ. Performed the experimental procedures: FB, MLSP, and FAB. Analyzed the data: FAB, FA, MLSP, MD, JC, GZ, and MBC. Drafted the paper: FAB, FA, and GZ. All authors read and approved the final manuscript.
